# A PCR blood test outperforms chromogranin A in carcinoid detection and is unaffected by proton pump inhibitors

**DOI:** 10.1530/EC-14-0100

**Published:** 2014-10-14

**Authors:** Irvin M Modlin, Harry Aslanian, Lisa Bodei, Ignat Drozdov, Mark Kidd

**Affiliations:** 1 Wren Laboratories, 35 NE Industrial Road, Branford, Connecticut, USA; 2 Yale University School of Medicine, 310 Cedar St, New Haven, Connecticut, USA

**Keywords:** biomarker, carcinoid, chromogranin A, gastroenteropancreatic, neuroendocrine, multigene transcript, NET, PCR, proton pump inhibitor

## Abstract

A critical requirement in neuroendocrine tumor (NET) management is a blood biomarker test that is sensitive, specific and reproducible. We evaluated a PCR-based 51-transcript signature to detect tumors, compared it with chromogranin A (CgA) and examined the confounding effect of proton pump inhibitors (PPIs), which cause falsely elevated CgA levels. The multigene signature was evaluated in two groups. Group 1: 125 prospectively collected NETs: gastroenteropancreatic NETs (*n*=91, including 42 pancreatic and 40 small intestinal), carcinoids of unknown primary (*n*=18) and other sites (*n*=16). Group 2: prospectively collected non-NET patients receiving PPIs (>1 month; dyspepsia, *n*=19; GERD, *n*=6; and pancreatitis, *n*=4) and 50 controls. All samples were analyzed by PCR (marker genes) and ELISA (DAKO–CgA). Sensitivity comparisons included *χ* ^2^, non-parametric measurements, and receiver operating characteristic (ROC) curves. Group 1: 123 NETs were PCR-positive (98.4%) compared with 50 (40%) CgA-positive (*χ* ^2^=97.3, *P*<10^−26^). Significant differences (*P*<0.001) were noted between pancreas: PCR 95% vs CgA 29.2% (*P*<10^−9^) and small intestine: 100 vs 58% (*P*<10^−4^). The multigene test was elevated in all grades (G1–G3), in both local and disseminated disease, and was not normalized by somatostatin analog therapy. It was also elevated in 97% of CgA normal NETs. Group 2: PPI administration increased CgA in 83% and CgA was elevated in 26% of controls. PCR values were not elevated in either group. PCR performance metrics were as follows: sensitivity 98.4%, specificity 100%, positive predictive value 100%, negative predictive value 97.8%, and the ROC-derived area under the curve (AUC) was 0.997. These were significantly better than CgA (all metrics <60%; AUC, 0.54; *Z*-statistic, 10.44, *P*<0.0001). A 51-panel multigene blood transcript analysis is significantly more sensitive than plasma CgA for NET detection and is unaffected by acid suppression therapy.

## Introduction

The National Cancer Institute (NCI) Neuroendocrine Tumor (NET) Summit conference proceedings of 2008 identified that a critical unmet need in the management of NET disease was the absence of a sensitive and specific set of tumor biomarkers (1). An accurate tumor marker is a critical tool in tumor management, because it establishes an uncertain diagnosis, offers a basis for individual prognostication, signals response to therapy, and identifies relapse. In classical terms, a high-quality tumor marker should represent a biologic attribute unique to the tumor cell or its local environment. Although this has proved manageable in a homogenous tumor population, the goal has been difficult to attain in gastroenteropancreatic NETs (GEP-NETs) as they comprise a heterogeneous group of cancers. Thus, tumor types range from histamine-secreting gastric ‘carcinoids’ to a mélange of pancreatic lesions (secreting glucagon, insulin, somatostatin, or vasoactive polypeptide – colloquially known as islet cell tumors), as well as small intestinal ‘carcinoids’ (secretin, serotonin, and a variety of tachykinins) and colorectal lesions (enteroglucagon, GLP1, and pancreatic polypeptide (PP)). The conundrum of identifying a global marker for NETs therefore has remained a substantial technical challenge.

A diverse variety of potential biomarkers has been proposed to be of utility in NET disease (2, 3, 4, 5). These include the constitutive neurosecretory protein chromogranin A (CgA) as well as individual secreted products, e.g. gastrin, serotonin, PP, neurokinin A, and VIP, or metabolic degradation products, e.g. urinary 5-HIAA. In general, these have proven to be relatively ineffective as biomarkers for a number of reasons. These include that they may only identify small subsets of lesions (specific, rare tumor types, e.g. VIPoma), the assays are not widely available (e.g. neurokinin A) or they are insensitive (e.g. PP in pancreatic NETs ∼50% sensitivity) (6), or there is a difficulty with the technique of collection (requires special diet and 24 h urine collection) (e.g. 5-HIAA). Furthermore, the overall performance metrics for these markers are low with <50% sensitivity with <30% specificity (2, 7, 8).

In general, CgA has, in the last decade, been widely utilized as the NET default biomarker (9). Its utility has been reflected in its generally broad NET recognition profile (10). Although elevated levels of CgA are generally considered to be sensitive, ∼60–90% accurate (11), it is ineffective as a first-line diagnostic for NETs (12). This reflects that measurements are non-specific (10–35% specificity) because CgA is elevated in other neoplasias, e.g. pancreatic and small-cell lung neoplasias and prostate carcinomas (13), and can be raised by a variety of cardiac and inflammatory diseases (14) as well as in renal failure (15). One of the commonest causes of spuriously elevated CgA levels is proton pump inhibitor (PPI) administration (10). A third of the USA population has been assessed to take acid-suppressive medications, e.g. PPIs (16, 17). Typically, more than 30% of persons over the age of 65 are estimated to use them (18, 19). The widespread use of PPIs is therefore a problematic confounder for a CgA biomarker assay.

Given the limited accuracy of the currently available biomarkers and the known limitations of single analyte measurements (20, 21), we developed a blood-based multianalyte NET-specific gene transcript analysis – the NETest. This is a robust, reproducible, PCR-based 51-marker peripheral blood signature (multigene test) with high sensitivity (85–98%) and specificity (93–97%) for the detection of gut NETs or ‘carcinoids’ (22, 23). The signature can identify all types of GEP-NETs, including small non-metastatic tumors, and significantly outperforms monoanalyte-based assays for detection (22, 24). In addition, the levels correlate with clinical status, e.g. stable or progressive disease (25). Based upon mathematical analyses of multianalyte methodology, this technique was determined to be superior to single-analyte assays in the detection of NETs (26).

The NETest conforms to a category of assays identified as Multianalyte Assays with Algorithmic Analyses (MAAAs), which include procedures that utilize multiple results derived from the assays of various types, including molecular pathology assays, e.g. breast cancer arrays (Mammaprint), fluorescent *in situ* hybridization assays, and non-nucleic acid-based assays (27). Algorithmic analyses, using the results of these assays as well as patient information (if available), are typically reported as a numeric score(s) or as a probability, often a risk probability (28), that can provide prognostic and predictive information, thereby aiding clinical decision-making (29). The strengths of MAAAs are the incorporation of multiple data sets as well as that these are typically undertaken by a single dedicated facility (30). Based on the need for an accurate test to assess NETs, we evaluated the specificity of the PCR-based test to detect tumors in comparison with CgA with particular reference to the confounding variable of PPI usage.

## Methods

### Sample collection

All samples were prospectively collected and analyzed according to a standard IRB protocol (Yale University: 6/17/2013) in accordance with the World Medical Association Declaration of Helsinki regarding the ethical conduct of research involving human subjects (22). All individuals from whom blood was obtained were present (6/2012–12/2013) at the School of Medicine out-patient clinics following an informed consent. The blood samples (5 ml) were collected in 9 mg K_2_EDTA tubes (BD Vacutainer Venous Blood Collection Tubes, BD Diagnostics, Franklin, NJ, USA). The aliquots of whole blood were stored at −80 °C within 2 h of collection (samples immediately stored on ice at 4 °C after sampling) per standard molecular diagnostics protocols for PCR-based studies (31). A second aliquot (2 ml) was spun (600 ***g***, 10 min) and the plasma collected for CgA ELISA using the DAKO Kit as described previously (22, 23, 32).

### Study groups

The clinical characteristics and details of the cases are included in Tables 1 and 2. These included Group 1: prospectively collected NETs (*n*=125) and Group 2: prospectively collected non-NET patients taking PPIs who underwent endoscopic retrograde cholangiopancreatography (ERCP) or endoscopic ultrasound (EUS) for upper gastrointestinal (GI) symptoms (*n*=29) at the Smilow Cancer Center, Yale New Haven Hospital. Group 1 included 91 GEP-NETs (gastric, *n*=3; duodenum, *n*=1; pancreas, *n*=41; small intestine, *n*=40; appendix, *n*=3; and colorectum, *n*=3), 18 with an unknown primary, and 16 non-GEP-NETs. Histopathologically, 51% were G1, 27% G2, and 12% G3. Disease was localized in 28% and was distant in 67%. No pathology and staging was available in 12 (10%) patients. Twenty-two percent were treatment naïve, while 26% were currently being treated with somatostatin analogs, and 9% (11 patients, all Zollinger-Ellison syndrome (ZES)) were on concomitant PPIs. None of the patients were noted to have hypertension or abnormal kidney function (review of medication lists and clinical history). Group 2 included pancreatic cysts, *n*=19; pancreatitis, *n*=4; and gastroesophageal disease (GERD), *n*=6. Fifty additional controls were included to measure the performance metrics of the multigene scores and CgA.

**Table 1:** Clinical characteristics of Group 1, NETs (includes GEP-NETs (*n*=91), CUP (*n*=18), and other sites (*n*=16)). Mean age 56.7 (range 25–82 years), gender: 40 males to 85 females, *n*=125.

**Tumor distribution**	**No.**	**Grade**			**Stage**		**Treatment**		**Current PPIs**
		G1	G2	G3	Local	Distant	Untreated	Current SSAs	
Gastric	3	0	1	2	3	0	3	0	0
Duodenum	1	1	0	0	1	0	1	0	0
Pancreas^a^	41	17	17	1	19	16	3	0	11
Small intestine	40	36	4	0	5	40	10	25	0
Appendix	3	3	0	0	1	2	1	0	0
Colorectal	3	2	1	0	2	1	1	1	0
CUP^b^	18	1	10	1	1	11	4	5	0
Lung	4	3	0	1	2	2	0	1	0
Ovary	1	0	1	0	0	1	0	0	0
Biliary tree	11^c^	1	0	10	1	11	4	0	0
Totals	125	64 (51%)	34 (27%)	15 (12%)	35 (28%)	84 (67%)	27 (22%)	32 (26%)	11 (9%)

CUP, carcinoid of unknown primary.

a Pathology data were only available for 35 of 41 pancreas NETs.

b Pathology data were only available for 12 of 18 CUPs.

c Ten were intrahepatic, one was extrahepatic.

**Table 2:** Clinical characteristics of Group 2.

**Characteristic**	**PPI users**^a^ (*n*=29)	**Controls** (*n*=50)	**Combined** (*n*=79)
Mean age (range) (years)	60 (46–79)	47.1 (28–75)*	50.5 (28–79)^†^
Gender (M:F)	11:18	23:27	34:45^‡^
GI pathology (C:G:P)	19:6:4	–	–

C, cyst; G, GERD; P, pancreatitis. *P<0.005 vs PPI (two-tailed Mann–Whitney *U* test); ^†^ *P*<0.03 vs NETs (two-tailed Mann–Whitney *U* test). ^‡^ *χ* ^2^=2.5, *P*=NS vs NETs (Fisher's exact test, two-tailed).

a Treatment includes lansoprazole (60 mg, *n*=1), omeprazole (5 mg, *n*=2; 20 mg, *n*=4; and 40 mg, *n*=6), pantoprazole (40 mg, *n*=4 and 60 mg, *n*=1), and rabeprazole (20 mg, *n*=1).

### PCR multigene test

A two-step manual technique protocol (RNA isolation with cDNA production and qPCR) was undertaken. The transcripts (mRNA) were isolated from 1 ml blood samples collected in an EDTA-coated tube using the Mini Blood Kit (Qiagen). The RNA quantity was 50 μl, the quality was >1.8 (A_260:280_ ratio); the analysis of the RNA pattern on electrophoresis (Agilent Technologies, Santa Clara, CA, USA) RNA Integrity Number (RIN) >5.0 (33). The standard Qiagen isolation protocol (heme/gDNA contamination not detected) with no modifications was used. cDNA was produced from 50 μl RNA using a high-capacity reverse transcriptase kit (Life Technologies: cDNA production 2000–2500 ng/μl) and stored at −80 °C. QPCR was carried out (384-well plate, HT-7900) with the cDNA (200 ng/μl) and 16 μl of reagents/well (Universal Master Mix II with UNG, Life Technologies, triplicate wells) (50 °C 2 min, 95 °C 10 min, then 95 °C 15 s, 60 °C, 60 s for 40 cycles) as described previously (22, 23). A NET score (0–8) is derived from the PCR data using MATLAB (R2011a, Mathworks, Natick, MA, USA) (25); a value ≥2 is a positive tumor score (22, 23, 25).

### Chromogranin A ELISA

CgA was measured using the DAKO ELISA Kit (K0025, DAKO North America, Inc., Carpinteria, CA, USA) (22, 23, 32). A cut-off of 19 U/l (DAKO) was used as the upper limit of normal. In preliminary studies, 75% of control samples (*n*=36) exhibited levels <14 U/l (22). An analysis identified that the recommended DAKO level of 19 U/l would result in a false positive of 3% (22). This cut-off was used in the current study.

### Statistical analyses

Sensitivity comparisons (*χ* ^2^, non-parametric measurements, and receiver operating characteristic (ROC) analysis) were made between the multigene test and plasma CgA (DAKO–ELISA) for the detection of NET. Both GraphPad Prism (LaJolla, CA, USA) and MedCalc (Ostend, Belgium) were utilized.

## Results

### Group 1

#### Prospectively collected NETs (*n*=125)

**Figure 1:**
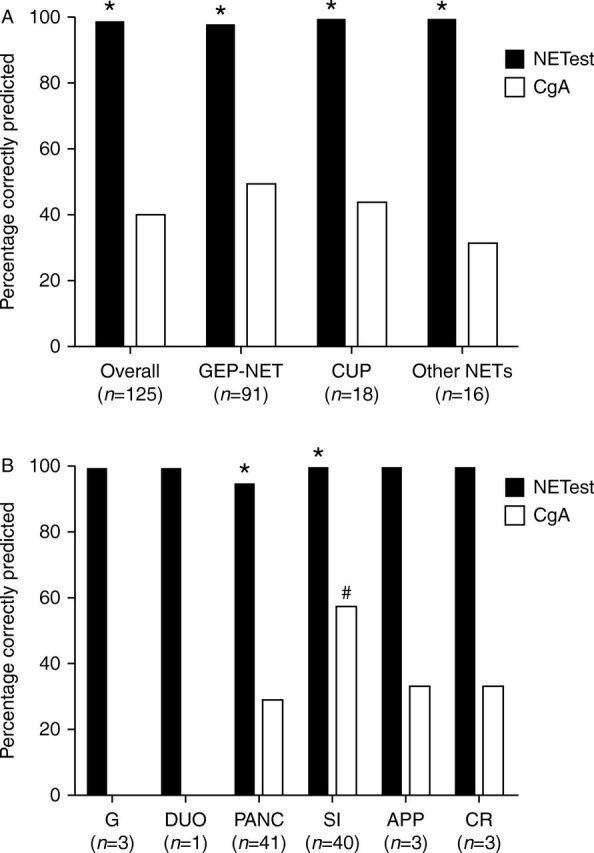
Accuracy of the multigene test compared with CgA for the detection of NETs (Group 1). (A) PCR analysis identified NETs in >98% compared with 40–50% with CgA. This was significantly better than CgA (*overall: *χ* ^2^=97.3, *P*<10^−26^; *GEP-NET: *χ* ^2^=52.3, *P*<10^−14^; *CUP: *χ* ^2^=11.2, *P*<0.0003; and *other NETs: *χ* ^2^=13.8, *P*<0.00006). (B) Assessment of primary site for GEP-NETs using PCR was >95%. CgA ranged from 0 to 56% correct cells. In the PANC and SI groups, the multigene test significantly out-performed CgA (*PANC: *χ* ^2^=35.1, *P*<10^−9^ and *SI: *χ* ^2^=19.1, *P*<10^−4^). Significantly more SI cases were CgA positive than in the PANC group (^#^ *P*<0.01). APP, appendix; CgA, chromogranin A; CR, colorectum; CUP, carcinoid of unknown primary; DUO, duodenum; G, gastric; GEP-NET, gastroenteropancreatic NET; PANC, pancreas; SI, small intestine.

One hundred and twenty-three (98.4%) had a PCR score ≥2 (positive test). In contrast, 50 (40%) exhibited an elevated CgA (Fig. 1A). The sensitivity of the PCR test for the detection of NETs was 98.4% (95% CI: 94.3–99.8%) vs 40% (95% CI: 31.2–49.4%) for CgA. This was highly significant (*χ* ^2^=97.3, *P*<10^−26^). A comparison between GEP-NETs (*n*=91) and CUP (*n*=18) or non-GEP-NETs (*n*=16) identified no significant differences in PCR-based detection (97.8–100%) between the different groups; CgA values for this group were (31–49.5%, Fig. 1A) with a significantly lower prediction rate (*P*<0.0005). An analysis of the individual GEP tumor sites (*n*=91) identified that the PCR test was positive in 95–100% of samples (gastric, 100%; duodenum, 100%; pancreas, 95%; small intestine, 100%; appendix, 100%; and colorectal, 100%); CgA positivity ranged between 0 and 58% (gastric, 0%; duodenum, 0%; pancreas, 29%; small intestine, 58%; appendix, 33%; and colorectal, 33%; Fig. 1B). A direct comparison of pancreatic and small intestinal NETs identified that PCR test values were almost identical (95 vs 100%, *P*=NS), but the CgA test was more often negative in the pancreas group (29.2 vs 58%, *χ* ^2^=5.5, *P*=0.014). Overall, the PCR values were elevated in 73 (97%) of NETs when CgA was in the normal range. This is of particular relevance in PNETs, where 27 (93.1%) of the 29 individuals with normal CgA exhibited an elevated multigene test.

#### Relationship with grade, stage, and treatment

The PCR score was positive in 123 (98%) of the samples irrespective of the grade (G1–G3) or stage (local or disseminated), whether the patient was treatment-naïve (27/27, 100% positive) or was on concomitant somatostatin analog (SSA) therapy (32/32). The two patients who did not have a positive score were both pancreatic: one was a 13 mm pancreatic NET, which had cystic features, and the second was neuroendocrine and subsequently identified to be a pancreatic metastasis from an ovarian NET. In both cases, CgA was normal. Eleven patients (9%) were on concomitant PPI therapy to control disease symptoms (ZES). All had positive PCR scores as well as CgAs. Elevated CgA levels exhibited no concordance with either grade or stage of disease or with SSA use.

### Group 2

#### Prospectively collected proton pump inhibitor-treated patients (*n*=29)

**Figure 2:**
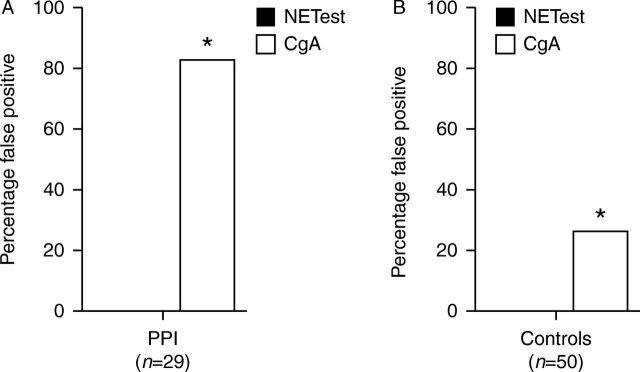
Evaluation of false-positives for PCR analysis compared with CgA (Group 2). (A) In the PPI group, the multigene test was 100% negative. However, CgA was elevated in >80% of PPI-treated individuals. This was statistically significant (*χ ^2^=37.6, *P*<10^−10^). (B) In the control group, none (0%) had an elevated PCR score. However, CgA levels were elevated in 13 (26%) (*χ ^2^=12.7, *P*<10^−3^). CgA, chromogranin A; PPI, proton pump inhibitor.

None (0%) had a positive PCR score ≥2 (positive test). In contrast, 24 (82.6%) exhibited an elevated (positive) CgA (ranging from 23 to 370 U/l, Fig. 2A). The false-positive CgA result was highly significant (*χ* ^2^=37.6, *P*<10^−10^).

#### Controls (*n*=50)

None (0%) had a PCR score ≥2 (positive test). In contrast, 13 (26%) exhibited an elevated CgA (ranging from 3.1 to 93.7 U/l, Fig. 2B). The false-positive result for CgA was highly significant (*χ* ^2^=12.7, *P*<10^−3^).

### Performance metrics

**Figure 3:**
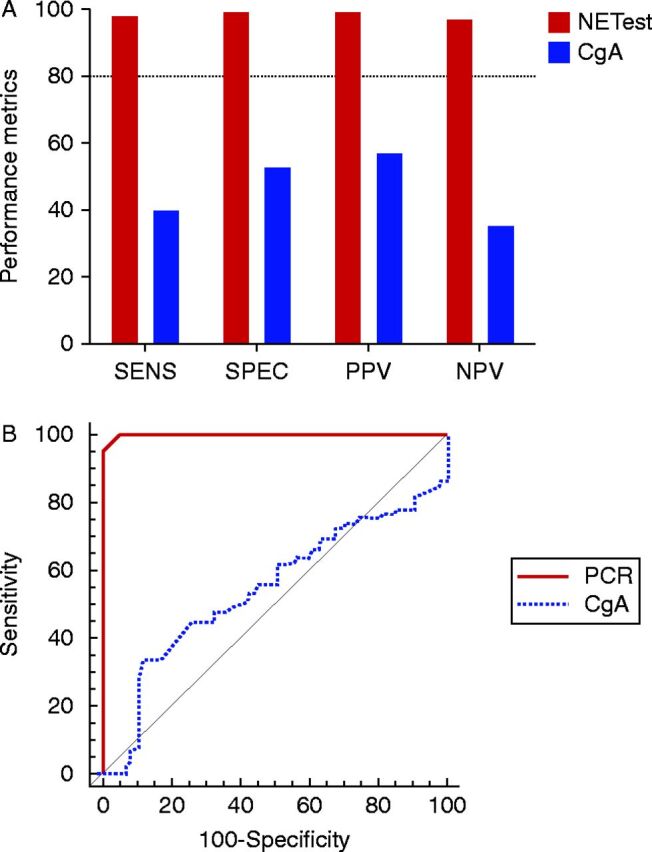
Performance metrics for the multigene test vs CgA. (A) The sensitivity, specificity, PPV, and NPV for the multigene test were all >80%. The metrics for CgA ranged from 39 to 57%. (B) Receiver operating characteristic (ROC) curves for PCR gene analysis compared with CgA. The AUC for PCR gene analysis was 0.997 and for CgA 0.54. This difference was highly significant (*Z*-statistic: 10.44, *P*<0.0001). The analyzed group included 125 NETs (all subtypes) and 79 controls (PPI treated, *n*=29 and controls, *n*=50). PCR, multigene test; CgA, chromogranin A; SENS, sensitivity; SPEC, specificity; PPV, positive predictive value; NPV, negative predictive value. The dotted line (A) represents 80% (standard cut-off level for biomarkers).

Overall, we examined the multigene test in 204 patients and controls, 61% (125) of whom can be considered as true positives (e.g. NETs). The remainder included GERD patients (*n*=29), none of whom had NETs (previous or current history) and 50 controls, ∼50% of whom were >50 years of age (none with past or current history of NETs). Overall, Group 1 was older (mean age 56.7 years (25–82)) than Group 2 (mean age 50.5 years (28–79), *P*=0.02), but no significant differences were noted between genders (33.6% (Group 1) and 43% (Group 2) men respectively). The performance metrics for differentiating a NET using the multigene test in these 204 patients and controls were: sensitivity 98.4% (95% CI: 94.3–99.8%), specificity 100% (95.9–100%), positive predictive value (PPV) 100% (97.0–100%), and negative predictive value (NPV) 97.8% (92.3–99.7%) (Fig. 3A). The AUC from the ROC curve for the multigene test was 0.997 (95% CI: 0.974–1.0, *P*<0.0001; Fig. 3B). For the CgA assay, these were as follows: sensitivity 40% (95% CI: 31.4–49.1%), specificity 53.2% (41.6–64.5%), PPV 57.5% (46.4–68%), and NPV 35.9% (27.2–45.3%). The AUC was 0.54 (95% CI: 0.47–0.62, *P*=0.32). A direct comparison of the AUCs (Hanley & McNeil (34)) identified that these were significantly different (difference between areas: 0.454, 95% CI: 0.37–0.54, *Z*-statistic=10.44, *P*<0.0001).

## Discussion

In NET disease, the identification of minimally detectable tumor activity or surrogates of tumor behavior is a key goal for early detection of alterations in tumor growth and metastasis, as well as for the assessment of therapeutic efficacy. To date, the use of either single-specific tumor products (e.g. serotonin, PP, and neuron specific enolase (NSE)) or a general marker of neuroendocrine secretion (CgA) has not met the rigorous criteria to be considered optimally effective in attaining these goals. We therefore utilized a PCR-based tool for the identification of GEP-NETs in peripheral blood to assess the diagnosis of NETs and evaluate the effect of common clinical situations, including PPI administration on test scores. In addition, we compared these results with CgA plasma measurements to assess comparability.

The PCR-based methodologies are widely considered as useful when the starting materials (e.g. circulating tumor cells or mRNA) are limited and require highly sensitive (35) and accurate tests (36). In the current study, we demonstrate that the PCR measurement of 51 multigene transcripts is highly accurate (98.4%) in the detection of NETs, and that it can detect a range of tumor types including GEP-derived tumors, and lesions with unknown primaries as well as bronchial and ovarian NETs. In particular, all grades were equally and effectively captured by the PCR test including 12 G3 tumors (gastric, pancreas, lung, and biliary tract) and no differences in a positive result were noted for local (including small benign insulinomas ∼12 mm diameter) or metastatic tumors (including liver and bone deposits). In particular, the use of SSAs was not associated with a negative test; all SSA-treated patients exhibited a positive PCR score. In addition, PCR analysis was positive in 97% of tumors when CgA levels are normal. This may be particularly useful in pancreatic NETs (<20% had elevated CgA levels in the current study). Two tumors not identified by the PCR test were both pancreatic: one a 13 mm lesion, identified on pathology to have cystic features and the second, a lesion subsequently identified to be an ovarian metastasis.

Overall, using a number of different analytic methods of performance (performance metrics, ROC curve analysis), the multigene test significantly outperformed CgA in the detection of NETs. The sensitivities and specificities in this independent data set (98–100%) are similar to those previously reported (85–98% sensitivity and 93–97% specificity) undertaken in different sample sets (22). The AUC (from the ROC curve) in the current study was 0.997, similar to that previously reported (95–98%) (22). CgA, in contrast, exhibited performance metrics <60% and the AUC from the ROC curve was 0.54. ROC curves for CgA generally range from 0.48 to 0.76 (12, 37, 38). These poor metrics have in recent times led a number of investigators to question its utility in NETs (12, 38).

While several commercially available and laboratory-developed assays have been developed (based on targeting different secretory fragments) (11), the calculated CgA level varies broadly between test platforms, all of which have varying sensitivities and specificities (39), and widely differing coefficients of variations (40). This reflects the highly heterogeneous antigen composite released by NETs following exocytosis, which comprise both the complete protein as well as a series of cleavage products that are smaller biologically active peptides (vasostatin I and II, chromacin, pancreastatin, WE14, parastatin, and catestatin) (8, 10). This is complicated by the fact that CgA processing varies between different neuroendocrine tissues, such that there is more extensive cleavage of CgA in pancreatic islets than in the adrenals, and different fragment profiles exist for each of the pancreatic alpha, beta, D, and PP cells (41). This has led to the development of different antibodies in the individual CgA immunoassays, which exhibit varying levels of detection (39, 42). A comparison of three commercial kits identified a range in sensitivities (67–93%) and specificities (85–95%) for the detection of NETs (39). In a second study of eleven assays, only four measured concentrations correlated with total CgA (42). Overall, the assays tend to be poorly correlated <30% (10). Irrespective of the kit type, no universally accepted CgA assay currently exists. Attempts at using more than one assay (43), or reconfiguring the CgA score to include other markers (44), only marginally increases the performance metrics and adds complex confounding variables (cost, inter-assay variation, and questions of interpretation). In a recent comprehensive analysis of this issue by Lewis & Yao (26), the authors have concluded that CgA could be supplemented or supplanted by PCR-based analysis of *NET* genes detectable in the blood transcriptome.

An effective circulating biomarker must exhibit three key technical attributes (45): i) the marker must be present in peripheral body fluid; ii) it must be easy to detect or quantify in assays that are both affordable and robust; and iii) its appearance must be associated as specifically as possible with a particular tumor, preferably in a quantifiable manner. In addition, the measurement of a biomarker should be accurate, reliable, and differentiate between normal and specific diseases. The performance metrics, including the AUC, should be >80% (46). The NET PCR transcript analysis meets these criteria. Conversely, using these criteria, CgA can be considered neither reliable nor robust, particularly given its confounding associations with a wide variety of non-NET conditions and commonly used medications. Unfortunately, clinicians are, to a large extent, unaware of such limitations and that CgA is only a moderately effective GEP-NET tumor biomarker.

The PPIs, in particular, represent a major iatrogenic cause of elevated circulating CgA levels. They are prescribed for GERD, esophagitis, acid hypersecretory states, peptic ulcers, and eradication of *Helicobacter pylori*. Overall, they are amongst the highest selling drugs worldwide (47, 48). Omeprazole therapy may result in CgA elevations that are in excess of 690 μg/l (mean 45±18 μg/l (normal range: 16–97 μg/l: CIS Kit (Bedford, MA, USA))) and can occur as early as 6 days after the first intake of the agent (16). CgA concentration is higher with PPI usage compared with the alternative class of acid suppressive agents, the histamine type 2 receptor antagonists (H_2_RA), given the more potent gastrin elevating capacity of the former group (49). As predicted, higher CgA levels are noted after long-term treatment (1–8 years) compared with mid-term (<1 year) or short (weekly/intermittent) treatment, reflecting the effect of sustained gastrin levels in increasing the proliferation of fundic ECL cells and may also cause G-cell hyperplasia (50, 51). In these circumstances, apart from elevated CgA, precursor neuroendocrine lesions of the fundus have been considered as a potential hazardous consequence of acid suppression (52). The widespread use of PPIs with consequent false positives (80% in the current study, but in up to 100% (39)) in measurement of this protein (and its fragments) amplifies the general clinical problem of diagnosis and management if decisions are based upon CgA as a NET biomarker. Thus, increases in CgA in such patients are usually consequent upon gastric and duodenal neuroendocrine cell hyperplasia (50, 51) (which is reversible following PPI withdrawal (52)). In contrast, the normal PCR values in these patients demonstrate that the molecular signature measures a tumor-specific event.

Peripheral blood biomarkers are considered to be a major resource for the prediction of disease and in aiding early diagnosis, as well as in setting standards (baselines) for the measurement of current or new remedies in disease treatment. The PCR multigene transcript analysis, unlike CgA, provides an accurate and sensitive measure of NET disease that is not affected by current acid inhibitory therapy. Furthermore, an additional advantage of the multianalyte marker set especially in NETs that are often indolent in their behavior (7) is that it can capture dynamic aspects of tumor biology. The cost of this MAAA test is currently unknown and undetermined since the insurance payers are the ultimate arbiter of reimbursement for molecular diagnostic tests. Economic analyses of MAAAs, however, demonstrate that development and clinical trials of biomarkers is cost-effective and provides both benefit as well as considerable cost-saving to society (approximately tenfold US$ benefit in terms of quality of life) (53). More pertinently, cancer center groups, such as the National Comprehensive Cancer Network (NCCN), have endorsed the use of high-performance biomarkers particularly in fields where there are no better alternatives (54). Irrespective of economic considerations, the high-sensitivity and specificity parameters of the NETest are consistent with an accurate and sensitive tool to identify NETs and assess disease progress using peripheral blood samples.

## Declaration of interest

The authors declare that there is no conflict of interest that could be perceived as prejudicing the impartiality of the research reported.

## Funding

This work was supported by Clifton Life Sciences.
